# Guillain–Barré Polyradiculoneuritis Developed in the Context of Lyme Neuroborreliosis in a 13-Year-Old Girl: A Case Report

**DOI:** 10.3390/children13040522

**Published:** 2026-04-09

**Authors:** Liliana Anghelina, Lucrețiu Radu, Magdalena Rodica Trăistaru, Cristian Gheonea, Rossy Vlăduț Teică, Anda-Lorena Dijmărescu, Adelina-Maria Anghelina, Ancuța-Ramona Camen

**Affiliations:** 1 Department of Pediatrics, University of Medicine and Pharmacy of Craiova, 200349 Dolj, Romania; 2 Department of Hygiene, University of Medicine and Pharmacy of Craiova, 200638 Dolj, Romania; 3 Department of Medical Rehabilitation, University of Medicine and Pharmacy of Craiova, 200349 Dolj, Romania; 4 Department of Radiology and Medical Imaging, University of Medicine and Pharmacy of Craiova, 200349 Dolj, Romania; 5 Department of Obstetrics and Gynecology, University of Medicine and Pharmacy of Craiova, 200349 Dolj, Romania; 6 University of Medicine and Pharmacy of Craiova, 200349 Dolj, Romania; adelina.anghelina@umfcv.ro; 7 Department of Occupational Medicine and Professional Diseases, University of Medicine and Pharmacy of Craiova, 200349 Dolj, Romania; ancuta.camen@umfcv.ro

**Keywords:** Guillain–Barré syndrome, Borrelia burgdorferi, Lyme neuroborreliosis, peripheral demyelination, functional recovery

## Abstract

**Highlights:**

**What are the main findings?**

• This report describes an extremely rare case of Guillain–Barré syndrome with acute flaccid paralysis and demyelinating neuropathy associated with Borrelia burgdorferi infection, a tick-borne spirochete and the causative agent of Lyme neuroborreliosis, in a 13-year-old girl with a family history of autoimmune disease.
• The case highlights the complexity of establishing the etiology of acute flaccid paralysis and demyelinating neuropathies, as well as the importance of prompt and appropriate treatment, with significant implications for both short- and long-term prognosis.

**What are the implications of the main findings?**

• Early diagnosis and prompt treatment of both conditions limited neurological damage and improved the short-term prognosis.
• Multidisciplinary management supported a favorable long-term recovery; after 7 months, the patient was able to walk with support and had no sensory or cognitive deficits.

**Abstract:**

**Background:** Guillain–Barré syndrome (GBS) is a rare, immune-mediated disorder affecting the peripheral nerves, often presenting with ascending muscle weakness and possible respiratory failure, usually following an infection. *Borrelia burgdorferi*, the causative agent of Lyme neuroborreliosis, is an uncommon trigger of GBS. **Case presentation:** We report the case of a 13-year-old girl with Lyme neuroborreliosis who developed sensorimotor GBS. She presented with progressive, symmetrical weakness, initially in the lower limbs and subsequently in the upper limbs, accompanied by absent deep tendon reflexes. Peripheral demyelination, confirmed in this case, is exceptionally rare. **Results:** Prompt diagnosis and appropriate treatment, together with rehabilitation measures, prevented further nerve damage. Seven months after onset, she was able to walk with support and had no sensory or cognitive deficits. **Conclusions:** Lyme neuroborreliosis can rarely trigger GBS with peripheral demyelination. Early recognition, timely intervention, and effective interprofessional collaboration limited the extent of nerve damage and promoted neurological recovery.

## 1. Introduction

Lyme disease is a bacterial infection caused by Borrelia spirochetes, which are introduced into the bloodstream through the bite of an infected tick. The infection can then spread to various tissues in the body, triggering a host immune response and leading to multisystem inflammation [1,2]. According to some studies, transmission of spirochetes within the first 12 h after a tick bite is rare [2]. Children are at higher risk of infection because they often do not report tick bites and may not comply with protective measures, such as covering exposed skin and using insect repellents [3]. According to the literature, Borrelia garinii, followed by Borrelia afzelii and Borrelia burgdorferi, is considered the most common etiological agent of neuroborreliosis in European adults; however, data on European children remain limited [4].

Lyme disease can present in three stages: early localized, early disseminated, and late disseminated. The clinical presentation varies depending on the stage of infection and may include erythema migrans, nonspecific constitutional symptoms, arthritis, carditis, and neurological manifestations known as neuroborreliosis [5]. Studies report that erythema migrans is absent in 20–30% of cases [6].

Lyme neuroborreliosis occurs in 10–15% of cases and most commonly presents with painful radiculitis, cranial nerve palsy (primarily facial palsy), and headache [7]. In children, the most frequent manifestations are facial nerve palsy and lymphocytic meningitis [8]. Peripheral demyelination associated with neuroborreliosis is extremely rare [9].

According to the literature, neurological manifestations in children with Lyme neuroborreliosis include headache (61%), fatigue (60%), cranial nerve paresis (59%), neck pain or stiffness (36%), fever >38 °C (30%), vomiting or nausea (23%), loss of appetite (18%), behavioral changes (12%), and vertigo (3%) [3].

According to the duration of neurological symptoms, Lyme neuroborreliosis is classified as early if symptoms have been present for less than six months, and late if for more than six months [10].

The European Federation of Neurological Societies has established criteria for definite and possible Lyme neuroborreliosis [10]. Definite Lyme neuroborreliosis (including all subtypes except advanced cases with polyneuropathy) requires the fulfillment of all three of the following criteria:

Neurological symptoms suggestive of Lyme neuroborreliosis (after exclusion of other potential causes).

Pleocytosis in the cerebrospinal fluid.

Intrathecal production of Borrelia burgdorferi sensu lato antibodies.

Possible Lyme neuroborreliosis is diagnosed when any two of the above criteria are met. If the third criterion is missing, Borrelia burgdorferi-specific Immunoglobulin G (IgG) antibodies must be detected in the serum after six weeks.

The two basic steps in establishing the diagnosis are ELISA screening and Western blot confirmation [11]. Lumbar puncture in children with Lyme neuroborreliosis is performed to exclude other diagnoses [12] and to detect pleocytosis [10].

When serological testing is positive, or when there is a high suspicion of neuroborreliosis despite the absence of antibodies in the blood, intrathecal production of antibodies, specifically non-specific Immunoglobulin M (IgM) for borreliosis, is evaluated. This is considered the gold standard for confirming the diagnosis of Lyme neuroborreliosis [13].

Computed tomography and magnetic resonance imaging of the brain or spinal cord may reveal focal abnormalities that are also present in other conditions, limiting their diagnostic value for Lyme neuroborreliosis [12].

Antibiotics with good central nervous system penetration are recommended for the treatment of Lyme neuroborreliosis. According to the National Institute for Health and Care Excellence guidelines, the following antibiotics are recommended for children aged 12 years and over [14]:

Lyme disease affecting the cranial nerves or peripheral nervous system:

• Doxycycline (oral): 100 mg twice daily or 200 mg once daily for 21 days;
• Amoxicillin (oral): 1 g three times daily for 21 days.

Lyme disease affecting the central nervous system:

• Ceftriaxone (intravenous): 2 g twice daily or 4 g once daily for 21 days;
• Doxycycline (oral): 200 mg twice daily or 400 mg once daily for 21 days.

Alternative treatment for central nervous system involvement:

• *Doxycycline* (oral): 100 mg twice daily or 200 mg once daily for 28 days;
• *Amoxicillin* (oral): 1 g three times daily for 28 days;
• *Ceftriaxone* (intravenous): 2 g once daily for 28 days.

Guillain–Barré syndrome is a monophasic, predominantly postinfectious, immune-mediated polyradiculoneuropathy that typically presents as an acute, symmetrical, and rapidly progressive flaccid paralysis of the lower and/or upper limbs (paraplegia or tetraplegia). This paralysis is accompanied by absent or diminished deep tendon reflexes and, in some cases, sensory disturbances [15]. Respiratory failure may occur when the lower cranial nerves (glossopharyngeal, vagus, and hypoglossal) or the nerves innervating the respiratory muscles are affected, necessitating mechanical ventilation in approximately 30% of cases [15].

Pathophysiologically, Guillain–Barré syndrome is triggered by an immune-mediated mechanism involving axonal and/or myelin disruption. Numerous infections have been associated with the etiology of Guillain–Barré syndrome, including Campylobacter jejuni, the most commonly identified pathogen, Epstein–Barr virus, Cytomegalovirus, Hepatitis E virus, Mycoplasma pneumoniae, Haemophilus influenzae, Influenza virus, and Zika virus [16,17]. A correlation with Lyme disease is attributed to Lyme neuroborreliosis, with rare cases reported in both adults and children [18,19]. According to a widely accepted hypothesis, Borrelia burgdorferi may trigger an autoimmune response. Components of this spirochete may act as antigens or form immune complexes, leading to the production of antiganglioside antibodies that may contribute to the development of Guillain–Barré syndrome in susceptible individuals [20]. The presence of ganglioside autoantibodies supports the diagnosis of inflammatory (autoimmune) neuropathy, although their absence does not exclude Guillain–Barré syndrome. These antibodies do not influence clinical management and are not useful for monitoring or prognosis [21].

The European Academy of Neurology and the Peripheral Nerve Society no longer support distinguishing between acute inflammatory demyelinating polyradiculoneuropathy and acute motor axonal neuropathy in Guillain–Barré syndrome [22]. In 2023, they established new diagnostic criteria that include essential criteria, clinical features, and other findings to support the diagnosis of Guillain–Barré syndrome [23].

Essential criteria:

• Progressive weakness of the upper and lower limbs;
• Absent or diminished deep tendon (osteotendinous) reflexes;
• Progression of clinical worsening not exceeding four weeks.

Clinical features supporting Guillain–Barré syndrome:

• Symmetrical neuropathy;
• Mild or absent sensory symptoms (compared to motor symptoms);
• Cranial nerve involvement (especially bilateral facial paralysis);
• Respiratory failure;
• Autonomic dysfunction;
• Recent gastrointestinal or respiratory infection (within the last six weeks);
• Back pain (interscapular or radicular).

Other findings supporting Guillain–Barré syndrome:

• Cerebrospinal fluid showing albuminocytologic dissociation;
• Electrophysiological findings confirming peripheral neuropathy.

These findings apply to typical Guillain–Barré syndrome, which presents as purely motor or sensorimotor involvement with symmetrical weakness in the arms and legs, accompanied by reduced or absent deep tendon reflexes.

In patients with suspected Guillain–Barré syndrome within the first week of symptom onset, reduced sensory nerve action potentials and/or compound muscle action potentials support a diagnosis of peripheral neuropathy. Absent H reflexes may suggest radiculopathy. The H reflex is the electrophysiological correlate of reflex muscle activation following electrical stimulation of afferent sensory fibers. Its absence is highly sensitive (95–100%) for Guillain–Barré syndrome [24,25], whereas its presence makes a diagnosis of Guillain–Barré syndrome unlikely [26,27].

Magnetic resonance imaging and ultrasound are not routinely employed in typical cases but may be considered when the diagnosis of Guillain–Barré syndrome is uncertain [23].

The current treatment for Guillain–Barré syndrome consists of administering intravenous immunoglobulin (IVIg) or plasma exchange (PLEX) as early as possible in the course of the disease. IVIg is possibly effective up to two weeks, and potentially up to four weeks, after disease onset, while PLEX may be effective up to four weeks. These treatments work by interrupting the processes that cause nerve damage, thereby preventing further injury and promoting functional recovery with reduced disability and a faster recovery rate. Randomized trials directly comparing IVIg and PLEX have shown no significant difference in treatment efficacy [28], and the latest guidelines do not recommend one treatment over the other [22]. IVIg treatment for Guillain–Barré syndrome administered at a dose of 2 g/kg over 5 days is associated with a lower risk of relapse compared to administration over 2 days [22]. Corticosteroids are not recommended for the treatment of Guillain–Barré syndrome. Intravenous methylprednisolone (IVMP) does not improve neurological outcomes [29,30], and combining IVMP with IVIg offers no additional benefit [31]. Oral corticosteroids may delay recovery or cause adverse effects [29,32].

Guillain–Barré syndrome is a treatable disease with a good potential for recovery: approximately 80% of patients regain the ability to walk independently, and more than 50% return to their previous baseline within one year [33]. The neuropsychological prognosis in children with Lyme neuroborreliosis is favorable; long-term neuropsychological disorders are not observed, in contrast to adults, who may experience cognitive impairment and persistent or recurrent neurological symptoms [34,35].

This article reports that Guillain–Barré syndrome can occur in the context of Lyme neuroborreliosis. Although extremely rare, cases of peripheral demyelination associated with Lyme neuroborreliosis have been documented in the literature. When rapidly evolving, ascending, symmetrical, and progressive neurological symptoms are present, thorough evaluation for both conditions is essential. Early diagnosis and appropriate treatment can prevent further nerve damage and support functional recovery. With timely therapy and a combination of medical and physiotherapeutic rehabilitation, both Lyme neuroborreliosis and Guillain–Barré syndrome generally have a favorable prognosis in pediatric patients.

## 2. Case Description

We present the case of a 13-year-old girl with no personal medical history, but with a family history of chronic conditions (maternal grandmother with rheumatoid arthritis and maternal aunt with systemic lupus erythematosus), who was hospitalized for lumbar pain radiating to the lower limbs, vomiting, and headache.

Two months ago, while on summer vacation at a camp, she noticed a round, erythematous lesion approximately 2 cm in diameter on the posterior region of her right thigh, which appeared to result from an insect bite. She subsequently began experiencing infrequent morning vomiting, mild headaches, fatigue, decreased appetite, and weight loss.

Seven days prior to admission, the patient presented with muscle pain in the posterior thighs, extending to the calves and lumbar region, accompanied by fever, dysphagia, odynophagia, mild headache, and nausea. She was treated with clarithromycin and symptomatic therapy, resulting in slight initial improvement. However, the lumbar pain subsequently intensified, radiating bilaterally to the lower limbs and accompanied by a headache and vomiting.

At the time of admission, the patient was afebrile with a slightly altered general condition. Her body weight was 41 kg, with a weak representation of adipose tissue, and the general clinical examination was otherwise normal. Oxygen saturation (SaO_2_) was 100%, heart rate 80 bpm, and blood pressure 120/70 mmHg. She showed no signs of meningeal irritation, balance disorders, gait abnormalities, or sensory deficits. Examination of the limbs revealed normal active and passive mobility, normal muscle strength, and present osteotendinous reflexes.

At the time of admission, the complete blood count revealed leukocytosis with neutrophilia and relative lymphopenia (Table 1).

The hemoglobin, hematocrit, inflammatory markers, blood gases/metabolic analysis, coagulation parameters, liver function tests, kidney function tests, and serum electrolyte panel showed normal values.

During the day of hospitalization, the patient’s general condition changed. She was hemodynamically and respiratorily stable, with an SaO_2_ of 100%, and was oriented to time and place. However, she reported intense pain in the lumbosacral spine and calves, along with paresthesias in both the upper and lower limbs. Subsequently, she developed gait and balance disturbances, cervical rigidity, and a positive Kernig’s sign.

The CT scan of the brain revealed no pathological changes. Lumbar puncture revealed clear cerebrospinal fluid with normal pressure, 56 lymphocytes/mm^3^, a positive Pandy reaction, an albumin level of 33 mg/dL, a chloride level of 760 mg/dL, and a glucose level of 66 mg/dL. Microscopic examination of the cerebrospinal fluid for Koch’s bacillus was negative.

Intravenous ceftriaxone treatment was initiated at 2 g twice daily, along with fluid and electrolyte rebalancing and symptomatic therapy, resulting in a favorable response within the first 24 h. Subsequently, the patient developed paravertebral muscle contracture, marked paresthesias, and severe muscle pain with functional impairment of the lower limbs, as well as worsening paresthesias in the upper limbs. Cervical rigidity increased, and the patient presented with photophobia and balance disturbances.

Neurological examination revealed bilateral limb muscle weakness, more pronounced in the lower limbs than in the upper limbs, along with reduced tendon reflexes in both upper and lower limbs. Cranial nerve examination was normal.

On the 10th day of admission, the patient presented with significant paravertebral muscle contracture, flaccid tetraparesis, absent osteotendinous reflexes, and decreased tactile sensitivity, more pronounced in the lower limbs. The patient was hemodynamically and respiratorily stable, oriented to time and space, conscious, and cooperative.

Neurological examination revealed a persistent motor deficit (plegia) in the lower limbs. Movement was observed only in the fingers of the upper limbs, with a paretic motor deficit more pronounced proximally than distally. On testing, only the bilateral bicipital reflex was present. The sphincters were continent, and cranial nerve function was normal.

Also, on day 10 of hospitalization, repeat laboratory testing showed persistent leukocytosis with neutrophilia and lymphopenia (Table 2).

All other investigations performed, including coagulation profile, blood gases and electrolytes, sodium (Na^+^), potassium (K^+^), total hemoglobin, hematocrit, blood glucose, C-reactive protein, fibrinogen, total proteins, albumin, urine analysis, liver function tests, creatine kinase, creatine kinase-MB, and lipase, showed normal values.

**Figure 1:**
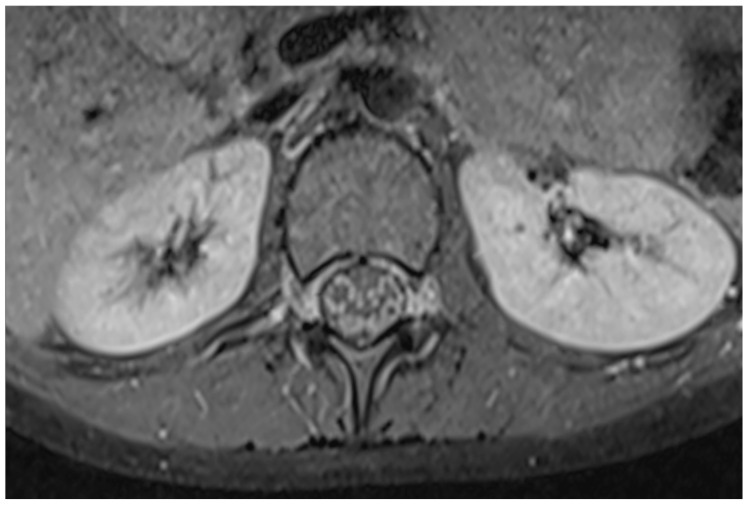
Post-contrast T1-weighted MRI of the lumbar spine demonstrating diffuse, symmetric enhancement of the anterior and posterior nerve roots of the cauda equina and lumbar spinal nerves. This imaging finding reflects breakdown of the blood-nerve barrier and increased vascular permeability secondary to inflammatory demyelinating radiculoneuropathy, as seen in Guillain–Barré syndrome. The enhancement is indicative of active inflammation and supports the diagnosis in the appropriate clinical context.

Magnetic resonance imaging (MRI) of the brain revealed no pathological changes. MRI of the lumbar spine showed diffuse thickening (maximum 2.8 mm) with a pronounced gadolinium-enhancing aspect of the anterior and posterior nerve roots at the level of the conus medullaris and cauda equina along the entire intradural length. Mild anterior epidural lipomatosis was observed at the L5-S1 level (maximum thickness 6.5 mm), extending caudally to the S2 level and causing complete occupation of the sacral spinal canal. The spinal cord demonstrated homogeneous signal intensity and was visible up to the T12 level. No vertebral compressions or focal bone lesions with suspicious MRI features were detected. These MRI findings are consistent with an acute polyradiculoneuritis lesion at the cauda equina and conus medullaris, which may be seen in Guillain–Barré syndrome (Figure 1).

Complex etiological investigations were performed:

Cultures and bacterial identification were conducted for methicillin-susceptible and methicillin-resistant Staphylococcus aureus, Streptococcus pyogenes, and beta-hemolytic Streptococcus groups C and G; all results were negative.

Cultures and bacterial identification were also performed for beta-lactamase-producing Enterobacteriales, carbapenemase-producing Enterobacterales, multidrug-resistant Acinetobacter baumannii, multidrug-resistant Pseudomonas aeruginosa, vancomycin-resistant Enterococcus faecium, and vancomycin-resistant Enterococcus faecalis; all results were negative.

Polymerase chain reaction tests were conducted for Influenza A/H1, A/H3, A/H1pdm, Influenza B, Respiratory Syncytial Virus type 2, Adenovirus, Enterovirus, Parainfluenza viruses 1–4, Metapneumovirus 3, Coronaviruses 229E, NL63, and OC43, SARS-CoV-2, Rhinovirus, and Bocavirus; all results were negative.

Serological tests (ELISA) were performed for IgM antibodies against Adenovirus, Coxsackie virus, Echovirus, Herpes simplex virus types 1 and 2, Cytomegalovirus (IgM and IgG), Epstein–Barr virus, Chlamydia pneumoniae, Mycoplasma pneumoniae, and Human Immunodeficiency Virus; all results were negative, except for IgM antibodies to Borrelia burgdorferi, which were positive.

Serum samples were collected for Borrelia burgdorferi IgM Western blot analysis, and cerebrospinal fluid was evaluated for intrathecal production of non-specific IgM antibodies to Borrelia; both tests were positive.

**Figure 2:**
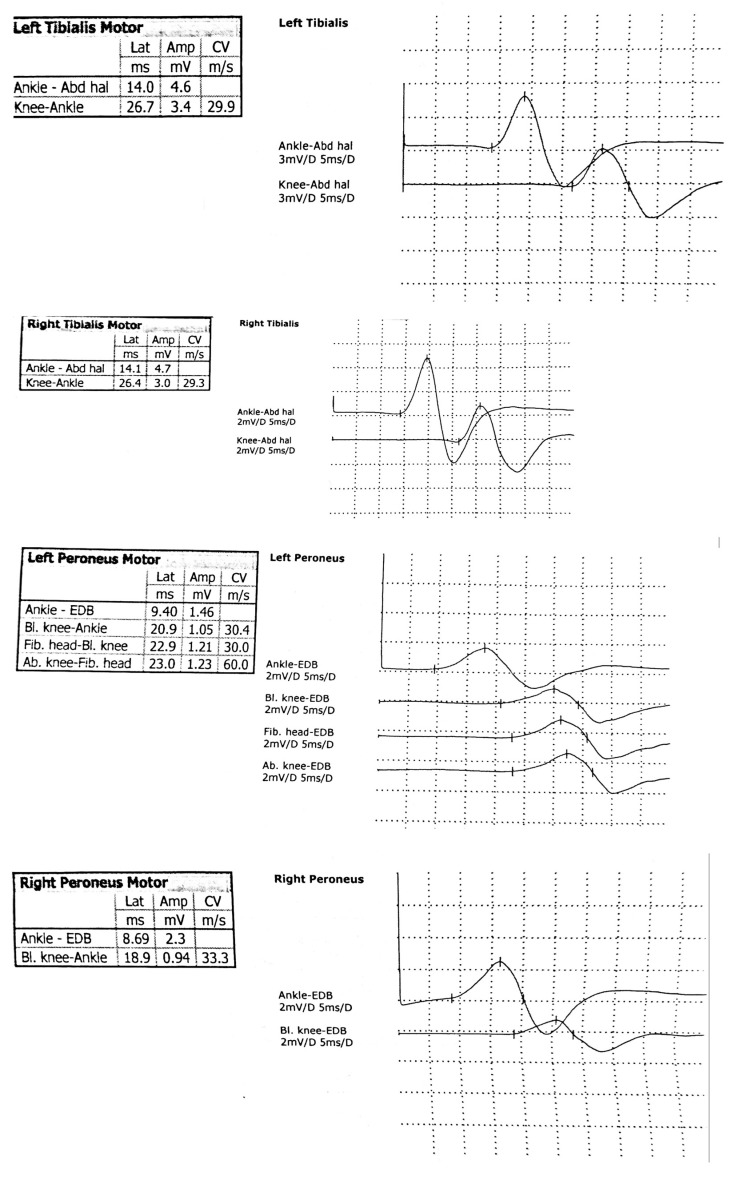
The motor nerve conduction study shows prolonged distal motor latency, normal amplitude, and reduced conduction velocity in both the tibial and peroneal motor nerves, bilaterally.

**Figure 3:**
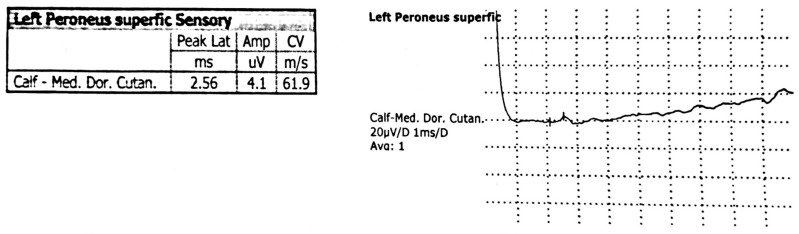
The sensory nerve conduction study of the left superficial peroneal nerve shows normal amplitude and conduction velocity.

**Figure 4:**
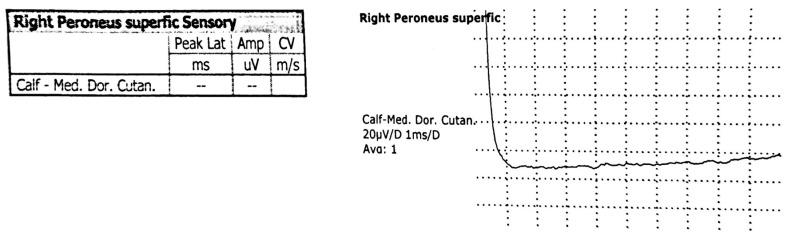
The sensory nerve conduction study shows absent conduction in the right superficial peroneal and left sural nerves.

Electromyography (Figure 2, Figure 3 and Figure 4), including needle electromyography, was performed and revealed a sensorimotor demyelinating impairment suggestive of Guillain–Barré syndrome.

Needle electromyography did not reveal any active denervation in the right and left tibialis anterior muscles during voluntary contraction; however, both muscles exhibited a neurogenic pattern characterized by prolonged duration and polyphasic motor unit potentials.

The therapeutic regimen included intravenous nonspecific human immunoglobulins (0.4 g/kg/day) for 5 days, ceftriaxone 2 g intravenously twice daily for 7 days, hydroelectrolytic rebalancing treatment, analgesics, and muscle relaxants. The clinical course was slowly favorable.

Complete blood count, inflammatory markers, blood gases, metabolic panel, coagulation parameters, liver and renal function tests, and serum ionogram all showed normal values.

At discharge, she was able to sit up with assistance and maintain the position but could not maintain an orthostatic (standing) position. Motor deficits were more pronounced in the lower limbs than in the upper limbs. Palmar-plantar paresthesias persisted.

Subsequently, she was treated with oral doxycycline, 100 mg twice daily, for 21 days and enrolled in a medical rehabilitation and physiotherapy program. After six weeks, the patient had normal active and passive mobility in both the upper and lower limbs. Segmental muscle strength in the lower limbs was slightly reduced on the right side, more so than on the left. Osteotendinous reflexes were absent in the lower limbs. The palmar-plantar paresthesias improved. There were no sensory or cognitive deficits. IgG anti-Borrelia antibodies (Western blot) were detected in the serum. A subsequent lumbar puncture revealed clear, normotensive cerebrospinal fluid with a Pandy reaction of 1+, low cellularity (15 nucleated elements/mm^3^), a slightly elevated protein level (97 mg/dL), and normal glucose levels. Borrelia burgdorferi-specific IgG antibodies (Western blot) in the cerebrospinal fluid were weakly positive.

A magnetic resonance scan of the spine showed no signal changes in the spinal cord or conus medullaris under native conditions.

**Figure 5:**
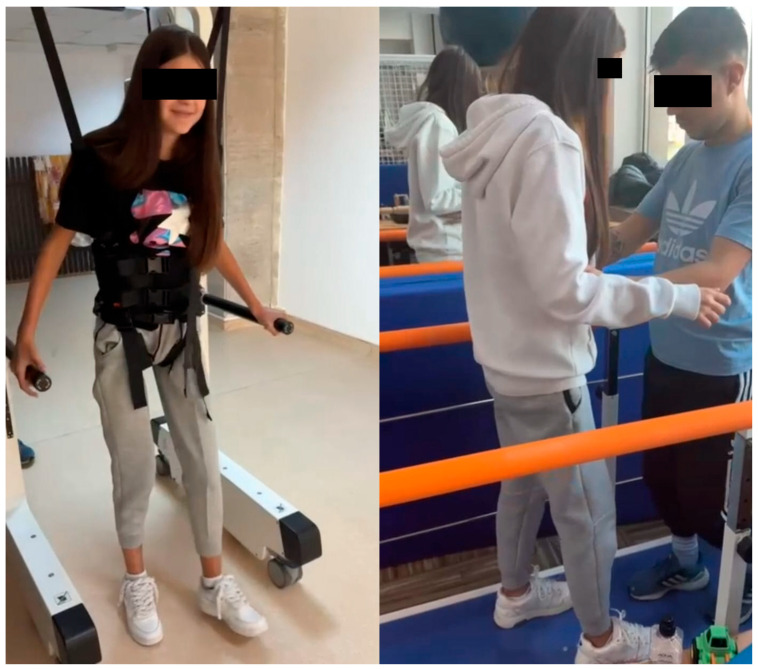
Rehabilitation program and physiotherapy results after seven months.

The child continued the medical rehabilitation and physiotherapy program, and seven months after the onset, she was able to walk with support (Figure 5).

## 3. Discussion

According to the literature, ticks are commonly found in rural and forested areas, gardens, and urban parks [36]. In this case, a 13-year-old girl noticed a lesion on the posterior region of her right thigh caused by a tick bite while attending a summer camp. Children are at higher risk of infection because they often do not report the bite and may not follow protective measures [3]. A detailed history revealed that the tick bite occurred two months prior to admission, during the summer camp. The patient subsequently developed neurological symptoms associated with neuroborreliosis in children, including infrequent vomiting, a mild headache, fatigue, decreased appetite, and weight loss [3]. Tick bites in children most commonly occur on the ears, neck, and especially the head, areas closest to the central nervous system, and are associated with a higher risk of developing Lyme neuroborreliosis [3], which most commonly presents as facial nerve paralysis and lymphocytic meningitis [8]. The highest incidence of Lyme borreliosis in children occurs between the ages of 5 and 9 years, peaking at age 7 [37,38]. One study found that boys with neuroborreliosis were generally older than girls [39].

Seven days prior to admission, the patient developed muscle pain in the posterior thigh, extending to the calves and lumbar region, accompanied by fever, dysphagia, odynophagia, mild headache, and nausea. She was treated with oral clarithromycin, which led to temporary improvement of symptoms, followed by worsening. Intravenous ceftriaxone was initiated upon admission. During hospitalization, her condition deteriorated rapidly, with increasing pain in the lumbosacral spine and calves, paravertebral muscle contracture, paresthesias in the upper and lower limbs, gait and balance disorders, cervical rigidity, and a positive Kernig’s sign.

Lumbar puncture revealed pleocytosis in the cerebrospinal fluid, which, together with the neurological symptoms, suggested possible neuroborreliosis according to the criteria of the European Federation of Neurological Societies [10]. Microscopic examination of the cerebrospinal fluid for Koch’s bacillus was negative.

Comprehensive investigations for an infectious etiology were conducted, including bacterial cultures, polymerase chain reaction (PCR) assays, and serological testing for a broad spectrum of bacterial and viral pathogens.

Serological testing with ELISA and Western blot for IgM antibodies against Borrelia burgdorferi in serum was positive [11]. Intrathecal production of non-specific IgM antibodies against Borrelia in the cerebrospinal fluid was also evaluated. This is considered the gold standard for confirming the diagnosis of Lyme neuroborreliosis [13], and the result was positive.

As the disease progresses, neurological symptoms become more pronounced in the lower limbs bilaterally, followed by rapid bilateral involvement of the upper limbs. Decreased tactile sensitivity is more prominent in the lower limbs, accompanied by paresthesias, reduced muscle strength, and initially diminished, then absent osteotendinous reflexes. Neurological examination revealed a persistent motor deficit (plegia) in the lower limbs and a paretic motor deficit in the upper limbs, more pronounced proximally than distally.

Magnetic resonance imaging of the lumbar spine revealed an acute polyradiculoneuritis-like lesion in the cauda equina and conus medullaris which can be seen in Guillain–Barré syndrome (Figure 1) and reflect disruption of the blood–nerve barrier. Electromyography (Figure 2 and Figure 4), including needle electromyography, revealed a sensorimotor demyelinating pattern, characteristic of Guillain–Barré syndrome, supporting the diagnosis of acute inflammatory demyelinating polyradiculoneuropathy, rarely encountered in neuroborreliosis [9]. Combined electrophysiological and magnetic resonance imaging findings confirm the diagnosis of Guillain–Barré syndrome and provide prognostic information: the predominance of demyelination and localized inflammation of the nerve roots suggests a favorable prognosis for recovery, while the presence of more extensive nerve damage would indicate the potential for a more prolonged course of the disease.

To exclude metabolic and electrolyte dysfunctions that may cause acute flaccid paralysis and demyelinating neuropathy, a series of laboratory tests was performed, including a complete blood count, C-reactive protein, fibrinogen, blood glucose, electrolytes, liver and kidney function tests, creatine kinase, creatine kinase-MB, and lipase. All results were within normal limits. The absence of inflammatory and muscle lysis markers ruled out infectious myositis. Magnetic resonance imaging of the brain and lumbar spine did not reveal any space-occupying lesions. Persistent leukocytosis with neutrophilia suggested an ongoing infectious process.

Rare cases of Guillain–Barré syndrome occurring in the context of Lyme neuroborreliosis have been reported in both adults and children [18,19]. The pathophysiological link between Borrelia burgdorferi infection and Guillain–Barré syndrome remains incompletely understood. It has been hypothesized that Borrelia burgdorferi may trigger an aberrant autoimmune response through molecular mimicry, leading to the production of antibodies that cross-react with peripheral nerve components. This process may result in demyelination contributing to the development of Guillain–Barré syndrome in susceptible individuals [20]. Genetic predisposition may represent an additional risk factor. The child’s family history includes chronic autoimmune diseases, such as a maternal grandmother with rheumatoid arthritis and a maternal aunt with systemic lupus erythematosus, suggesting increased susceptibility to immune dysregulation and the development of post-infectious autoimmune complications.

According to the European Academy of Neurology and the Peripheral Nerve Society, the clinical manifestations are consistent with sensorimotor Guillain–Barré syndrome, characterized by symmetrical muscle weakness in both lower and upper limbs, decreased tactile sensitivity, more pronounced in the lower limbs, and the absence of deep tendon reflexes [23].

The therapeutic regimen included intravenous administration of nonspecific human immunoglobulins at a dose of 0.4 g/kg/day for 5 days [22], ceftriaxone 2 g intravenously twice daily for 7 days [14], supportive treatment to restore fluid and electrolyte balance, as well as analgesics and muscle relaxants.

In the present case, rapid diagnosis and the establishment of adequate therapy halted neurological progression. Cranial nerve function was normal, and there was no respiratory failure, which can develop in up to 30% of cases [15].

The clinical course was slowly favorable, and upon discharge, the patient was able to sit up with assistance and maintain her position but could not sustain an upright (standing) posture. She exhibited a more pronounced motor deficit in the lower limbs than in the upper limbs, along with palmar-plantar paresthesia.

She subsequently underwent treatment with oral doxycycline, 100 mg twice daily for 21 days, according to the National Institute for Health and Care Excellence guidelines [14], and was enrolled in a medical rehabilitation and physiotherapy program.

After six weeks of evolution, Borrelia burgdorferi-specific IgG antibodies (detected by Western blot) were found in the serum, thus fulfilling the criteria for Lyme neuroborreliosis as defined by the European Federation of Neurological Societies [10]. Additionally, Borrelia burgdorferi-specific IgG antibodies (Western blot) in the cerebrospinal fluid were weakly positive. Lumbar puncture revealed clear, normotensive cerebrospinal fluid with a 1+ Pandy reaction and low cellularity (15 nucleated elements/mm^3^). Magnetic resonance imaging of the spine showed no abnormalities in the spinal cord or conus medullaris. The clinical course was favorable, with normal active and passive mobility in both the upper and lower limbs. Segmental muscle strength in the lower limbs was slightly reduced, and palmar-plantar paresthesias improved.

The long-term neuropsychological prognosis in children with Lyme neuroborreliosis is favorable [34,35], and Guillain–Barré syndrome is a treatable disease with potential for recovery [33]. In the case presented, seven months after onset, the child has no sensory or cognitive deficits and can walk independently.

## 4. Conclusions

Borrelia burgdorferi, a spirochete introduced into the bloodstream by a tick bite, can cause Lyme neuroborreliosis and trigger an autoimmune response leading to the development of Guillain–Barré syndrome. Peripheral demyelination associated with neuroborreliosis is extremely rare in both adults and children. Guillain–Barré syndrome in the context of Lyme neuroborreliosis should be suspected when the patient’s condition does not improve with antibiotic therapy and when they develop ascending, rapidly progressive, symmetrical muscle weakness of the lower and upper limbs, along with reduced or absent deep tendon reflexes. Prompt treatment of both conditions is crucial for preventing further nerve damage and supporting functional recovery. Neuropsychological rehabilitation requires a multidisciplinary approach, focusing on helping the child regain independent mobility and return to their initial functional state.

## Figures and Tables

**Table 1:** Tests with abnormal values.

Class/Parameter	Value
White blood count	12.99 × 10^3^/µL
Neutrophils	81.3%
Lymphocytes	11.7%

**Table 2:** Tests with abnormal values.

Class/Parameter	Value
WBC	12.58 × 10^3^/µL
Neutrophils	83.31%
Lymphocytes	10.62%

## Data Availability

Further anonymized data related to this study are available from the corresponding author upon reasonable request.

## Abbreviations

The following abbreviations are used in this manuscript:

AIDP	Acute inflammatory demyelinating polyradiculoneuropathy
AMAN	Acute motor axonal neuropathy
*B. burgdorferi*	*Borrelia burgdorferi*
CNS	Central nervous system
CSF	Cerebrospinal fluid
CT	Computed tomography
ELISA	Enzyme-linked immunosorbent assay
EFNS	European Federation of Neurological Societies
EAN	European Academy of Neurology
GBS	Guillain–Barré syndrome
HIV	Human Immunodeficiency Virus
IgG	Immunoglobulin G
IgM	Immunoglobulin M
IVIg	Intravenous immunoglobulin
IVMP	Intravenous methylprednisolone
MRI	Magnetic resonance imaging
MDD	Major depressive disorder
NICE	National Institute for Health and Care Excellence
PCR	Polymerase chain reaction
PLEX	Plasma exchange
PMI	Post-mortem interval
SaO_2_	Oxygen saturation
WBC	White blood cell count
